# A Diagnostic-Oriented Screening Scale for Anxiety Disorders: The Center for Epidemiologic Studies Anxiety Scale (CESA)

**DOI:** 10.3389/fpsyg.2020.00957

**Published:** 2020-05-26

**Authors:** André Faro, William W. Eaton

**Affiliations:** ^1^Department of Psychology, Federal University of Sergipe, São Cristóvão, Brazil; ^2^Department of Mental Health, Bloomberg School of Public Health, Johns Hopkins University, Baltimore, MD, United States

**Keywords:** anxiety screen, anxiety disorders, agoraphobia, social phobia, blood-illness phobia, panic disorder

## Abstract

**Objectives:**

This paper introduces a new diagnostically oriented screening scale for anxiety disorders, the Center for Epidemiologic Studies Anxiety scale (CESA), designed in parallel to the revised Center for Epidemiologic Studies Depression scale (CESD-R). In this study, the CESA was used as a diagnostic screening tool for detecting the presence of anxiety disorder symptomatology ascertained by a clinical psychiatric evaluation based on the DSM-5 criteria. The CESA is designed to provide an overall evaluation of anxiety as well as to screen for four important anxiety disorders (agoraphobia, social phobia, blood-illness phobia, and panic disorder).

**Methods:**

The test sample was composed of 80 adults seeking treatment for mental problems in a general psychiatric clinic. We assessed the receiver operating characteristic (ROC) curve of the CESA in comparison to the psychiatric interview.

**Results:**

The main findings suggest that the CESA is useful for screening for anxiety in general (alpha coefficient of 0.83), as well as for the four common anxiety disorders. The criterion validation confirmed a high level of compatibility between the CESA and the psychiatric evaluation.

**Conclusion:**

This is the initial report regarding the CESA and future research will focus on specific aspects of criterion validity for each disorder.

## Introduction

Anxiety and depression are two of the most common mental disorders worldwide (Steel et al., 2014; Craske et al., 2017). According to the World Health Organization, there are about 300 million cases of one or both diagnoses, with lifetime prevalence rates of 4.4% for depression and 3.6% for anxiety disorders (World Health Organization [WHO], 2017). According to the Global Burden of Disease (GBD) study, depressive disorders and anxiety disorders are, respectively, third and ninth when it comes to the leading conditions of Years Lived with Disability (YLD) (Vos et al., 2016).

The Center for Epidemiologic Studies Depression scale (CESD) (Radloff, 1977) is one of the most widely used instruments for measuring depressive disorders worldwide (Van Dam and Earleywine, 2011). The CESD was designed prior to the publication of the third edition of the Diagnostic and Statistical Manual of Mental Disorders, in 1980, and failed to include some of the symptoms necessary for a complete diagnosis. Consequently, the revised version (CESD-R) was published in 2004 to fit the DSM-IV criteria for major depressive disorder (Eaton et al., 2004). Both the CESD and CESD-R are widely employed in studies around the world, in particular for screening population-based and primary care samples, which may include a range of physical conditions (Van Dam and Earleywine, 2011; Nabbe et al., 2017). The CESD-R is available on the web as a mobile app^1^. The website includes translations of the CESD-R into 11 languages.

Anxiety disorders are conditions that share characteristics of disproportionate fear, cognitive, and/or somatic responses, which sometimes include dysfunctional behaviors (American Psychiatric Association [APA], 2013; Craske et al., 2017). Globally, the current prevalence of any anxiety disorder is estimated to be 7.3% in the general population (Baxter et al., 2012). Currently, Brazil has the highest (9.3%) and Vietnam the lowest (2.2%) prevalence rates worldwide (World Health Organization [WHO], 2017). Anxiety disorders show common symptomatology even when manifested in different contexts, which contributes to frequent diagnostic overlap within the major classification of anxiety problems (Bystritsky et al., 2013; Bandelow and Michaelis, 2015; Craske et al., 2017).

There are several instruments for screening anxiety symptomatology as a general clinical condition, like the Beck Anxiety Inventory (BAI) (Beck and Steer, 1990), the State-Trait Anxiety Inventory (STAI) (Balsamo et al., 2013), and the Overall Anxiety Severity and Impairment Scale (OASIS) (Norman et al., 2011). These instruments generally have good psychometric features, but they fail to measure the diversity of clinical conditions associated with different manifestations of anxiety disorders.

Interviews such as the DIS (Robins et al., 1989) and CIDI (Kessler and Ustun, 2004), and structured examinations such as the SCID (Spitzer et al., 1992) and SCAN (Wing et al., 1990) are available, which diagnose a range of specific anxiety disorders. These instruments have the advantage of making a diagnosis, not just generating a probability of a positive diagnosis, as with screening scales such as the CESD-R; but they require much more training to administer, and more time from the respondent, than brief screening scales like the CESD-R. There are a number of instruments that screen for the possibility of a positive diagnosis for specific anxiety disorders but do not purport to make a diagnosis: for example, the Generalized Anxiety Disorder-7 (GAD-7) (Spitzer et al., 2006) with 7 items; the Panic and Agoraphobia Scale (PAS) with 13 items (Bandelow, 1995); the Mobility Inventory for Agoraphobia (MIA) with 32 items (Chambless et al., 2011); the Agoraphobia Scale with 20 items (Öst, 1990); the Panic Disorder Severity Scale (PDSS) with 7 items (Shear et al., 1997); the Liebowitz Social Anxiety Scale (LSAS-CA) with 24 items (Liebowitz, 1987); and the Social Phobia Inventory (SPIN) with 17 items (Connor et al., 2000). The CESA was designed to be concise and practical, as with the CESD and CESD-R. A distinctive aspect of the CESA is that, as well as providing an overall anxiety score, it screens for the presence of four common anxiety disorders in a single brief screening tool: agoraphobia, social phobia, blood-illness phobia, and panic disorder.

Agoraphobia is defined as exaggerated anxious and fear responses to open or closed places or to daily situations such as being in a queue or a crowd (American Psychiatric Association [APA], 2013). Grant et al. (2006) found lifetime prevalence rates ranging from.05 to.17% in the United States general population. Despite having one of the lowest prevalences among anxiety disorders, agoraphobia is included in the CESA due to its high impairment and comorbidity with other anxiety disorders (Bonham and Uhlenhuth, 2014).

The main characteristics of social phobia are fear of being criticized in social interactions, such as public speaking experiences (American Psychiatric Association [APA], 2013). Social phobia has been included because of its high prevalence (Kessler et al., 2012; Stein et al., 2017) and important consequences of social avoidance. Using data from 26 countries, Stein et al. (2017) identified a 12-month and lifetime prevalence rates of 2.4 and 4.0%, respectively (see also Ohayon and Schatzberg, 2010).

The CESA includes blood-illness phobia because avoidance of doctors or injections by these individuals may have important consequences for public health (Oosterink et al., 2009; Armfield, 2010; Potter et al., 2014; Wani et al., 2014). The Epidemiologic Catchment Area (ECA) study found that the lifetime prevalence of blood-injection phobia ranged from 0.7% for men to 3.3% for women (Bracha et al., 2007).

Panic disorder is the recurrent experience (often unexpected) of panic attacks associated with persistent worry about having another attack (American Psychiatric Association [APA], 2013). From a worldwide sample, de Jonge et al. (2016) estimated a lifetime prevalence for panic disorder of 1.7%. Lifetime prevalence rates of panic disorder in the Americas, the United States, and Brazil, respectively, were 2.2, 4.7, and 1.7% (de Jonge et al., 2016).

The CESA has an advantage over prior scales because it screens for the possibility of four distinct disorders, requires no training to administer, and requires only a few minutes of the respondent’s time. As with the CESD-R, this will be advantageous in population surveys and also in primary health care (Vermani et al., 2011). This study introduces the CESA and performs a validation using a clinical psychiatric evaluation of the presence of any anxiety disorder as the single criterion diagnosis. We also present the algorithm for converting response patterns in the CESA to probabilistic categories for diagnoses.

## Method

### Participants

The sample was comprised of 80 adults by the convenience criterion, who were seeking treatment for mental problems in a general psychiatric clinic located in Sergipe (Brazil). Patients of both sexes were invited to fill out the CESA while they were waiting for the psychiatrist appointment (private care, out-of-pocket). Interviews were conducted after discussing and agreeing to take part in the study. The data collection was done 2 days per week and lasted 4 months (4-month institutional authorization). Cases of psychosis, drug dependence, intellectual or communication disabilities; patients younger than 18 or older than 70 years old; and those who declared they felt uncomfortable to engage in the research were excluded.

### Instruments

The *Center for Epidemiologic Studies Anxiety Scale (CESA)* contains 20 items, fits on a single sheet of paper, and requires 3–5 min to complete (see Supplementary Material 1). The items were created after reviewing prior scales of anxiety, with a view toward simplicity of phrasing and parallel response patterns to make the scale easy to understand and respond to. Part A lists common triggers of three types of phobias with questions about individual experiences in the 6 months before the interview. Items 1, 2, and 3 are devised to detect agoraphobia; items 4 and 5 are aimed at social phobia; and items 6 and 7 are for blood-illness phobia. The items on phobia were chosen to reflect what were regarded as the most common sources of phobic fear. Part B includes physical and psychological symptoms that are in the diagnostic criteria for panic disorder, but also relevant to anxiety disorders in general. Each respondent was asked questions in part B even if they had a low level of response (i.e., value of 1) to any one of the symptoms in part A. Part C contains one question that measures the frequency and crescendo quality of panic attacks.

Parts A and B are answered in a four-point ordinal scale of frequency. In part A, the responses range from 0 (No, never) to 3 (Yes, and I avoided the situation almost all of the time) and the final score of the seven items ranges from 0 to 21 points. Part B has response alternatives comprising 0 (No, never), 1 (Yes, sometimes), 2 (Yes, often), and 3 (Yes, almost every time)—thus, with 12 questions, part B might reach 36 points. Part C has four alternatives related to duration of reactions to the symptoms in part B: 0 (No, Never); 1 (Once or Twice); 2 (Three times or more); or 3 (Many times) (see Supplementary Material 1).

In this study, the CESA was translated into Brazilian Portuguese (see Supplementary Material 2). First, the English version was translated and back-translated into Brazilian Portuguese. Two bilingual translators evaluated the process and produced a final first-version translation. Then the original and the initial translated versions were sent to seven bilingual researchers in the field of mental health [Ph.D. in health psychology (2), developmental psychology (2), psychiatry (1), psychometrics (2)] in order to evaluate the any possible language, theoretical, or cultural differences between both versions and the DSM-5 criteria for any anxiety disorder. After small adjustments on the writing and theoretical compatibility of the items in English and Brazilian Portuguese, a pretest conducted with 30 undergraduate students in psychology revealed no issues in relation to comprehension about the instructions, items, and scale answers.

The criterion validation for this study consisted of psychiatric diagnoses by one of two psychiatrists using open-ended interviews based on DSM-5 (American Psychiatric Association [APA], 2013) for detection of anxiety disorders (see DSM-5, pp. 189–190). From individual interviews and analysis of the presence of significant symptoms related to anxiety disorders (i.e., fear, avoidance, anxiety, behavioral disturbances, specific cognitive ideations, and physical symptoms), the psychiatrists stated a general positive or negative diagnosis of any anxiety disorder for each patient.

### Procedures

This study was approved by the Human Research Ethics Committee of the Federal University of Sergipe (Brazil), and conducted in compliance with the 1964 Declaration of Helsinki and later addenda to the Declaration. All participants were voluntary and agreed with the research terms by the signature of the informed consent. The CESA and the psychiatric interview were performed in separate sessions, blind to each other but on the same day, with an interval of nearly 1 h between filling of the CESA and the psychiatrist’s interview.

### Data Analysis

We used SPSS (version 21) to calculate scores and to perform descriptive and inferential statistical analysis. Analysis of sensitivity and specificity of the CESA in relation to the diagnosis of any anxiety disorder, as well as the examination of the cutoffs, was done using the receiver operating characteristic (ROC) curve (Habibzadeh et al., 2016). Cronbach’s alpha, McDonald’s omega, and Guttmann’s Lambda 6 were used to estimate the internal consistency reliability of the CESA (Hair et al., 2014).

## Results

### Sample Profile

The sociodemographic and health characteristics of the sample are summarized in Table 1. The final sample comprised 77.5% females with an average age of 38.8 years (SD 13.02). Most subjects declared their skin color as either *Parda* (mixed race) or black (46.3%), with white (45.0%) as the second most self-referred group. A majority reached the level of college graduation (73.8%), and most subjects were affiliated with some religion (75.0%), employed (72.5%), and in a marital relationship (55.0%). Sixteen percent of the participants declared having received a diagnosis of a depressive disorder sometime during their life, 27% a diagnosis of any anxiety disorder, and 50% a diagnosis of both anxiety and depressive disorders. Table 1 includes percentages of the demographic groups with values of the CESA equal to or above the cutoff. These values are not always what might be predicted on the basis of prior research (i.e., construct validity) because the sample is not drawn from the general population, but rather from those seeking treatment for psychiatric disorders.

**TABLE 1:** Descriptive statistics of sociodemographic and health data of 80 subjects in a general psychiatric clinic.

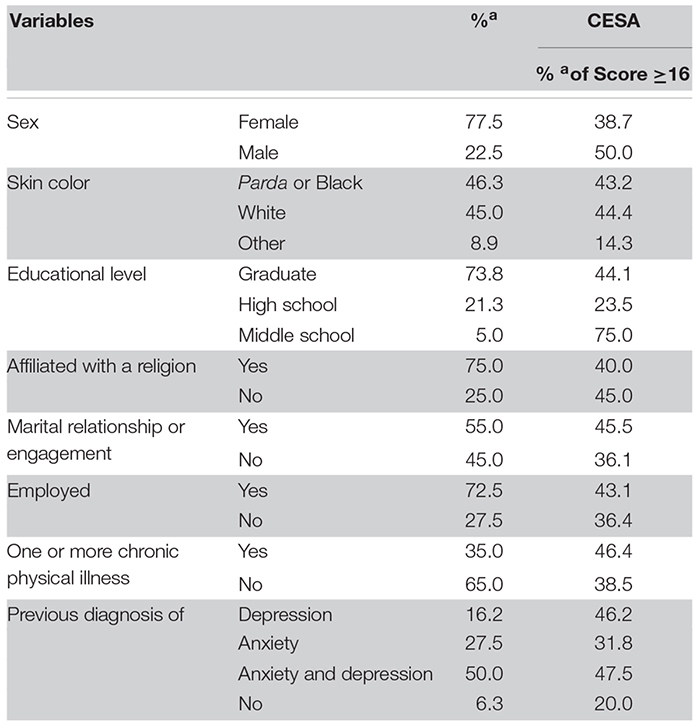			

### Descriptive Statistics of the CESA Items

Table 2 describes the answers to all CESA items. In part A, 6.3% of the subjects did not respond positively to any item. Among the participants who declared that they had experienced some trigger to phobia, the item that received the highest number of strong positive answers (i.e., 26% with score 3: “I avoided the situation almost all the time”) was fear of speaking in public. Other phobia items were reported by little more than 10% at that level, with the exception of fear of blood or injection, which was reported by 7.5% of subjects at that level. In item 1, the most frequent answer was at level 2 (“Yes, and sometimes I avoided the situation”—20.0%). The two most frequent negatives (No, never) were “afraid of seeing blood or getting a shot” (item 6, 75.0%) and “afraid of seeing a doctor or dentist” (item 7, 76.3%). These two items correspond to blood-illness phobia.

**TABLE 2:** Descriptive statistics of the CESA items in the full sample (*n* = 80).

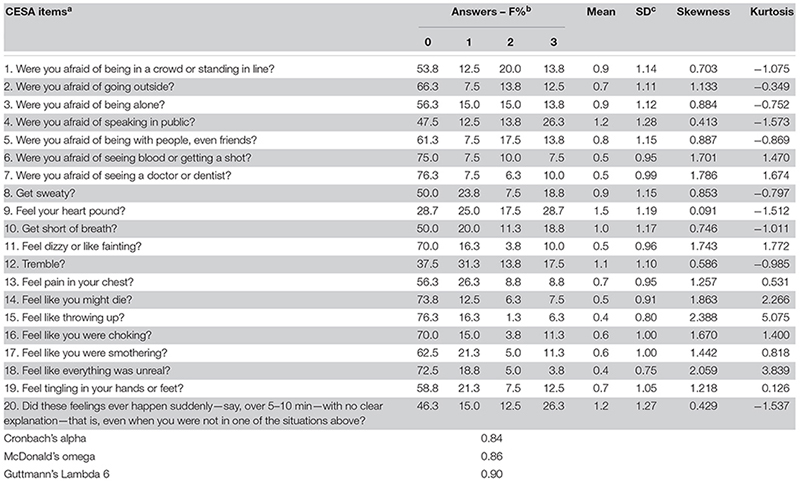								

In part B, the most common symptom answered as 3 (Yes, almost every time) was feeling the heart pound (28.7%) and trembling (17.5%). The less common symptoms were feeling like throwing up and feeling like they might die, with 76.3 and 73.8% negative answers, respectively. In relation to the crescendo aspect of panic (part C), 26.3% said that the symptoms happened suddenly many times in the last 6 months, and 46.3% denied that it ever happened that way (level 0).

### Criterion Validity Analysis and Diagnostic Characteristics

The average of the CESA’s score was 15.7 (SD 10.69) and the median was 14 (*Minimum* 0–*Maximum* 53). Measures of reliability showed satisfactory indices of the internal item’s consistency (Cronbach’s alpha = 0.84, McDonald’s omega 0.86, and Guttmann’s Lambda 6 = 0.90). The ROC curve of the CESA presented an area under the curve (AUC) of 0.90 (*p* < 0.001) at a score of 16 (Sensitivity = 0.58 and Specificity = 0.96; Youden Index = 0.54). Using the algorithm presented in Supplementary Material 3, with the cutoff of 16 (as in the original CESD) and requiring at least one answer at level 3, the prevalence of any anxiety disorder was 76.3%.

Table 3 shows the composition of the categorical diagnoses provided by the CESA for the 61 respondents who screened positively for any anxiety disorder (Table 3). The algorithm for arriving at the case definitions is also presented in Supplementary Material 3. First, we estimated the phobia classification based on the score of the items in part A. Items were added from each phobia and the parameter used for the categorization was the final score. For example, in the analysis of agoraphobia (items 1, 2, and 3), those participants who had score 1–3 were classified as possible cases (34.4%), scores 4–6 as probable (11.5%), and 7–9 as highly probable cases of agoraphobia (36.1%). Negative cases were those who scored 0 (18.0%). For social phobia (items 4 and 5), 29.5% were negative (score 0) and 44.3% were highly probable (score 5 or 6). Cases of blood-illness phobia were less common than the other phobias: only 19.7% were classified as highly probable (score 5 or 6) and 54.1% were negative (score 0). For panic disorder, persons with no crescendo quality to their anxiety (no to item 20) were considered negative (85.2%). Possible cases (6.6%) were those with occasional crescendo (response of 1 on item 20) as well as a sum of 8 or more on items 8–19, including 1 or more answers at the level of 3 in part B. Probable cases (1.6%) were those with the crescendo aspect present three or more times (response at level 2), as well as a sum of 10 or more and 2 or more answers in part B at the level of 3; highly probable cases (6.6%) had responses of 3 on item 20, as well as a sum of 12 or more and 3 or more answers 3 on items 8–19.

**TABLE 3:** Descriptive statistics and diagnostic classification of the CESA among 61 respondents screening positive for any anxiety disorder.

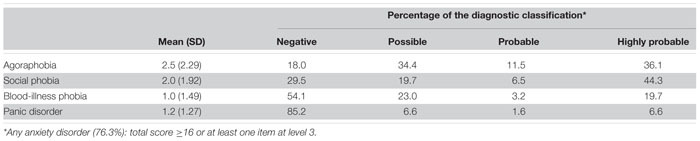					

## Discussion

This paper reports the creation and initial validation of a new scale to screen for the trait of anxiety as well as four specific anxiety disorders. The four disorders were chosen for their importance in terms of prevalence and associated impairment. We have reached four main conclusions. First, the content validity of the CESA, consisting of the choice of the items based on the DSM-5, is strong. Second, the CESA showed high internal consistency, as might be expected based on the comorbidity of the anxiety disorders (Bystritsky et al., 2013; Bandelow and Michaelis, 2015). Third, there is suggestive evidence that it can identify probable cases of four specific anxiety disorders correctly. Fourth, the criterion validation confirmed a high level of compatibility between the CESA and the psychiatric evaluation as to the presence of any anxiety disorder. The criteria of 6 months for the classification of an anxiety disorder followed the diagnostic orientations in the DSM-5, and this characteristic refers to the ability of the CESA in catching not only recent episodes of anxiety or even transient responses to stressful events. This is important because it allows the analysis of the enduring symptoms according to the criterion for the diagnosis of anxiety disorders. This time interval for detection reveals another strength of the CESA, since other scales for anxiety disorders usually limit the appearance of the symptoms to recent weeks, for example: OASIS to the past week (Norman et al., 2006); GAD-7 to the last 2 weeks (Spitzer et al., 2006); and the Mini-SPIN to the last week (Seeley-Wait et al., 2009). We believe that the CESA will be useful for screening in population-based and primary care research due to its brevity and capacity to integrate symptoms, triggers, and probability levels in a single measurement for different diagnostic possibilities.

### Limitations and Conclusions

Several limitations of this investigation should be discussed. First, because we used a small clinical sample, selected by convenience criteria and with time restrictions, larger systematic clinical and community-based samples are important to assess the more general performance of the CESA.

Second, due to constraints of time and the institutional setting that required limitation to a single appraisal and psychiatric evaluation, the ROC curve analysis was applied only to the generic diagnosis of anxiety disorders. Future studies should perform criterion validations for all disorders investigated through the CESA. It is suggested to use mainly structured interviews, such as SCID-5, SCAN, CIDI and M.I.N.I., because they are highly reliable parameters for the diagnosis of mental disorders involving examination by a clinician as opposed to self-report. It is also recommended to estimate deeper levels of agreement in the inter-rater evaluation (e.g., symptom by symptom), which could be facilitated through the use of standardized instruments, such as those mentioned above.

Third, also due to the small sample size, we did not examine the distribution of key demographic variables in the study of anxiety such as sex and age. Thus, future investigations should evaluate demographic, familial, and stress-related differences in the score and probability of the four possible diagnoses using the CESA. In addition, another aspect for further research is to perform different psychometric assessments with this new scale (i.e., concurrent, discriminant, and predictive validity). We hope that the CESA will be a valuable new open-access tool for screening of anxiety disorders in psychiatric and primary care clinics, as well as population-based surveys of the prevalence of anxiety disorders.

## Data Availability Statement

The datasets for this manuscript are not publicly available because the authors are still working on additional analysis related to the research project. Requests to access the datasets should be directed to AE, andre.faro.ufs@gmail.com.

## Ethics Statement

The studies involving human participants were reviewed and approved by Human Research Ethics Committee of the Federal University of Sergipe (Brazil). The patients/participants provided their written informed consent to participate in this study.

## Author Contributions

AF and WE designed the study, conducted the analysis, and wrote the manuscript. Both authors contributed to manuscript revision and read and approved the submitted version.

## Conflict of Interest

The authors declare that the research was conducted in the absence of any commercial or financial relationships that could be construed as a potential conflict of interest.

## Footnotes

1 http://cesd-r.com
