# Supplementary material for: A Diagnostic-Oriented Screening Scale for Anxiety Disorders: The Center for Epidemiologic Studies Anxiety Scale (CESA)
Source: Front Psychol. 2020 May 26;11:957. doi: 10.3389/fpsyg.2020.00957 (PMC7265241; doi:10.3389/fpsyg.2020.00957)
Supplement: Supplementary file 1 [file Table_1.pdf]

Supplementary Material 1

Original Version of the CESA

CENTER FOR EPIDEMIOLOGIC STUDIES ANXIETY SCALE - CESA

	0	1	2	3
**A. Over the last six months:**	No, never	Yes, but never enough to change what I was planning or doing	Yes, and sometimes I avoided the situation	Yes, and I avoided the situation almost all the time
1. Were you afraid of being in a crowd or standing in line?				
2. Were you afraid of going outside?				
3. Were you afraid of being alone?				
4. Were you afraid of speaking in public?				
5. Were you afraid of being with people, even friends?				
6. Were you afraid of seeing blood or getting a shot?				
7. Were you afraid of seeing a doctor or dentist?				
**B. When you were in these situations, did you...**	No, Never	Yes, sometimes	Yes, often	Yes, almost every time
8. Get sweaty?				
9. Feel your heart pound?				
10. Get short of breath?				
11. Feel dizzy or like fainting?				
12. Tremble?				
13. Feel pain in your chest?				
14. Feel like you might die?				
15. Feel like throwing up?				
16. Feel like you were choking?				
17. Feel like you were smothering?				
18. Feel like everything was unreal?				
19. Feel tingling in your hands or feet?				
	No, never	Once or twice	Three times or more	Many times
**C. 20. Did these feelings ever happen suddenly—say, over 5-10 minutes-- with no clear explanation—that is, even you were not in one of the situations above?**				
