# Supplementary material for: A Diagnostic-Oriented Screening Scale for Anxiety Disorders: The Center for Epidemiologic Studies Anxiety Scale (CESA)
Source: Front Psychol. 2020 May 26;11:957. doi: 10.3389/fpsyg.2020.00957 (PMC7265241; doi:10.3389/fpsyg.2020.00957)
Supplement: Supplementary file 2 [file Table_2.pdf]

Supplementary Material 2

Brazilian Portuguese Version of the CESA

CENTRO PARA ESTUDOS EPIDEMIOLÓGICOS – ESCALA DE ANSIEDADE (CESA)

	0	1	2	3
**A. Nos últimos 6 meses, você…**	Não, nunca	Sim, mas nunca o suficiente para mudar o que eu estava planejando ou fazendo	Sim e às vezes eu evitei a situação	Sim e evitei a situação quase o tempo todo
1. Teve receio de estar em uma multidão ou em pé numa fila?				
2. Teve receio de ir à rua?				
3. Teve receio de ficar sozinho(a)?				
4. Teve receio de falar em público?				
5. Teve receio de estar com as pessoas, mesmo que sejam amigas?				
6. Teve receio de ver sangue ou tomar uma injeção?				
7. Teve receio de ir ao médico ou dentista?				
**B. Tendo passado ou não por essas situações, nos últimos 6 meses você...**	Não, nunca	Sim, às vezes	Sim, frequentemente	Sim, quase a todo o tempo
1. Suou demais?				
2. Sentiu o coração bater muito rápido?				
3. Ficou com dificuldade de respirar?				
4. Sentiu tontura e como se fosse desmaiar?				
5. Ficou tremendo?				
6. Sentiu uma dor ou aperto no peito?				
7. Sentiu que iria morrer?				
8. Ficou com vontade de vomitar?				
9. Sentiu como se estivesse engasgando e ficando sem ar?				
10. Sentiu como se estivesse ficando sufocado(a)?				
11. Sentiu como se tudo não parecesse ser real?				
12. Sentiu formigamento nas mãos ou nos pés?				
	Não, nunca	Uma ou duas vezes	Três vezes ou mais	Muitas vezes
**C. 20. Essas sensações (Parte B) já aconteceram de repente – digamos, por 5-10 minutos – sem um motivo claro – isto é, mesmo você não estando em alguma das situações acima (Parte A)?**				
