# Supplementary material for: A Diagnostic-Oriented Screening Scale for Anxiety Disorders: The Center for Epidemiologic Studies Anxiety Scale (CESA)
Source: Front Psychol. 2020 May 26;11:957. doi: 10.3389/fpsyg.2020.00957 (PMC7265241; doi:10.3389/fpsyg.2020.00957)
Supplement: Supplementary file 3 [file Table_3.pdf]

Supplementary Material 3

Center for Epidemiologic Studies Anxiety Scale - CESA

Algorithm for Diagnosis

Anxiety Score (Any Anxiety Disorder)
Anxiety Score: sum of items 1-20 (range 0-60)
Negative (“not anxious”): 0-15 total score, with no items at level 3
Positive (“anxious”): total score equal or above 16, or at least one item at level 3

Agoraphobia (items 1-3)
Negative: sum of zero
Possible: sum of 1-3 (no score of 3)
Probable: sum of 4-6 (no score of 3)
Highly probable: sum of 7-9

Social Phobia (items 4 and 5)
Negative: sum of zero
Possible: sum of 1-2 (no score of 3)
Probable: sum of 3-4 (no score of 3)
Highly probable: sum of 5-6

Blood-Illness Phobia (items 6 and 7)
Negative: sum of zero
Possible: sum of 1-2 (no score of 3)
Probable: sum of 3-4 (no score of 3)
Highly probable: sum of 5-6

Panic Disorder
Negative: 0 in item 20
Possible: 1 on item 20, sum of items 8-19 $\geq$ 8, 1 or more answer = 3 on items 8-19
Probable: 2 on item 20, sum of items 8-19 $\geq$ 10, 2 or more answer = 3 on items 8-19
Highly Probable: 3 on item 20, sum of items 8-19 $\geq$ 12, 3 or more answers = 3 on items 8-19
